# Obesity and Multiple Sclerosis Susceptibility: A Review

**DOI:** 10.29245/2572.942x/2016/7.1064

**Published:** 2016

**Authors:** Milena A. Gianfrancesco, Lisa F. Barcellos

**Affiliations:** Division of Epidemiology, School of Public Health, University of California, Berkeley, Berkeley, CA, USA

**Keywords:** Multiple sclerosis, Obesity, Body mass index, Autoimmune disease, Epidemiology, Susceptibility

## Abstract

Several studies conducted around the world over the last decade have demonstrated that early childhood and adolescent obesity are significant risk factors for MS susceptibility. This association has been largely confirmed in females, while evidence supporting a strong role for obesity and risk of MS in males has been mixed. Further, interaction between increased body mass index and genetic as well as environmental factors in MS susceptibility has been proposed, and evidence of a causal relationship has recently been established. In this review, we discuss findings supporting the significant association between obesity and MS, as well as identify areas for future investigation.

## Introduction

Multiple sclerosis (MS) is an autoimmune disease of the central nervous system that affects over 400,000 Americans and approximately 2.3 million people worldwide^1,2^. It is characterized by the presence of inflammation, neurodegeneration, and demyelinating lesions of white and gray matter. Health care costs in the U.S. associated with MS range from $8,500 to upwards of $52,000 per patient year^3^. Both genetic and environmental factors have been implicated in MS etiology. Several genetic variants, including the human leukocyte antigen (HLA)-DRB1*15:01 allele within the major histocompatibility complex (MHC)^4^ and 110 non-MHC variants^5^, have been identified. Environmental risk factors associated with MS onset include exposure to tobacco smoke, Epstein-Barr Virus or infectious mononucleosis (IM), low levels of vitamin D, and most recently, obesity^6^.

## Obesity as a Risk Factor for Multiple Sclerosis

Obesity is a current public health problem around the world; approximately 35% of adults in the U.S. are obese^7,8^. Further, obesity has more than doubled in children and quadrupled in adolescents over the past 30 years^9,10^. Described below are several studies over the last decade demonstrating that early childhood and adolescent obesity are significant risk factors for MS susceptibility. Therefore, the increasing prevalence of obesity could potentially be contributing to higher rates of MS in children and adults.

The first comprehensive study to examine the relationship was in two cohorts of women from the Nurses’ Health Study (NHS) I and II (n=238,371)^11^. The researchers found that women with a BMI ≥ 30 kg/m^2^ at age 18 had a 2.25-fold increased risk of developing MS compared to those with a BMI in the normal range (18.5 to < 21 kg/m^2^) after adjusting for age, latitude at age 15, race/ethnicity, and smoking. In contrast, there was no association between baseline BMI and MS risk. The study also compared silhouette images of body type and found a similar twofold-increased risk of MS for women reporting a larger body size at age 20 compared to those who reported a thinner body size (adjusted RR=1.96, 95% CI 1.33, 2.89). Estimates for larger body type at ages 5 and 10 trended toward significance. These findings contrasted with an earlier study that noted an inverse association between increased BMI and odds of MS (OR=0.76, 95% CI 0.61, 0.95), though the sample size in this case-control study was small, and BMI was self-reported at diagnosis^12^.

Confirming findings from NHS, researchers in Sweden utilized a population-based study of MS cases (n=1,571) and controls (n= 3,371) and found that self-reported weight at age 20 was ~3% higher in cases^13^. Further, after adjusting for age, sex, residential area, ancestry and smoking, individuals with a BMI ≥ 30 kg/m^2^ had two-fold increased odds of MS (OR=2.1, 95% CI 1.5, 3.0); the association was significant in both females and males.

Additional studies have replicated a two-fold increased risk of MS as a result of obesity, including data derived from population-based samples from Norway and Italy^14^. In this case-control study, participants were asked to describe their perceived body size at various ages using silhouettes similar to those used in NHS. After adjusting for age, smoking and sun exposure, researchers found a significant trend of increased risk of MS with increasing body size from age 15–25 in the Norwegian group, with the highest estimate for body size at age 25 (OR males = 2.21, 95% CI 1.09, 4.46; OR females=1.43, 95% CI 0.90, 2.27). A similar but non-significant pattern was observed in the Italian group.

A study within the Kaiser Permanente, Northern California membership also examined the relationship between increased BMI and MS, but for the first time additionally controlled for genetic factors, including 110 established non-MHC MS risk variants^15^. Similar estimates were found, including a two-fold increased risk of MS in females with a BMI ≥ 30 kg/m^2^ (OR=2.15, 95% CI 1.18, 3.92) in one’s twenties. No significant association was observed in males. In contrast to the findings from NHS, a significant association was shown for self-perceived body size at age 10 (‘little/very overweight’ vs. ‘just about right’) and MS (OR = 1.50, 95% CI 1.15, 1.97) in females.

A role for childhood obesity and risk of both pediatric and adult-onset MS had previously been confirmed in two studies^16, 17^. Results demonstrated that obesity was significantly associated with an increased risk of pediatric-onset MS or clinically isolated syndrome (CIS) (n=75)^16^. Those who were moderately obese had 1.28 times the odds of developing MS/CIS, and those who were extremely obese had 2.10 times the odds of developing disease. When stratified by sex, the association was significant in females, but not males. Further, extremely obese girls had over three times the odds of developing disease compared to those at normal weight.

In a Danish prospective study, school records from more than 300,000 individuals were utilized to examine BMI in childhood and risk of adult-onset MS^17^. Researchers found that a one-unit increase in a BMI z-score from ages 7–13 was consistently associated with a significant increased risk of MS (HR=1.17–1.20) in girls. Boys also had an increased risk, but the results were significant only for ages 8–10 (HR=1.14–1.15). Percentile based comparisons of males and females together reflected an increased risk of MS ranging from 1.56–1.81 when comparing BMI > 95th percentile to < 85th percentile.

A recent study contrasts with these findings by demonstrating an increased risk of adolescent, but not childhood obesity^18^. While self-reported weight and height were used to determine BMI at age 20, silhouette images were utilized to characterize self-reported body size at age 10. Individuals with larger silhouettes demonstrated 1.5 increased odds of MS after controlling for age, sex, residential area, ancestry, smoking, and sun exposure. However, this association was diminished after additionally controlling for BMI at age 20. Associations were similar in males and females. The researchers also observed a significant association between adolescent obesity amongst females and earlier age of onset (p<0.0001). This finding is in agreement with a small case-only study of women (n=184), which reported being overweight at age 25 was associated with significantly earlier MS onset (~5 years) compared to not being overweight^19^.

In summary, while there is a general consensus that obesity in young adulthood, particularly from ages 18–25, is associated with MS susceptibility, the association between childhood obesity and MS is less clear. Differences in findings may be attributed to the use of silhouettes in some studies to assess body size, and the fact that overweight individuals have been shown to have a more a favorable perception of body silhouettes^20^, potentially biasing results towards the null. Nonetheless, childhood obesity is strongly associated with both adolescent and adult obesity, with almost two-thirds of children in the highest BMI quartile remaining in the highest BMI quartile during young adulthood^21^. Therefore, targeting obesity during childhood may be important in reducing risk of MS in the population. It has been estimated that eliminating childhood obesity could prevent approximately 15% of MS cases^6^. While the exact window of susceptibility may vary based on an individual’s genetics and environmental factors, childhood, in addition to adolescence, remains a particularly vulnerable period of exposure for MS risk, as has been suggested for other environmental factors such as sunlight exposure^22^.

## Genetic and Environmental Interactions

**Figure 1:**
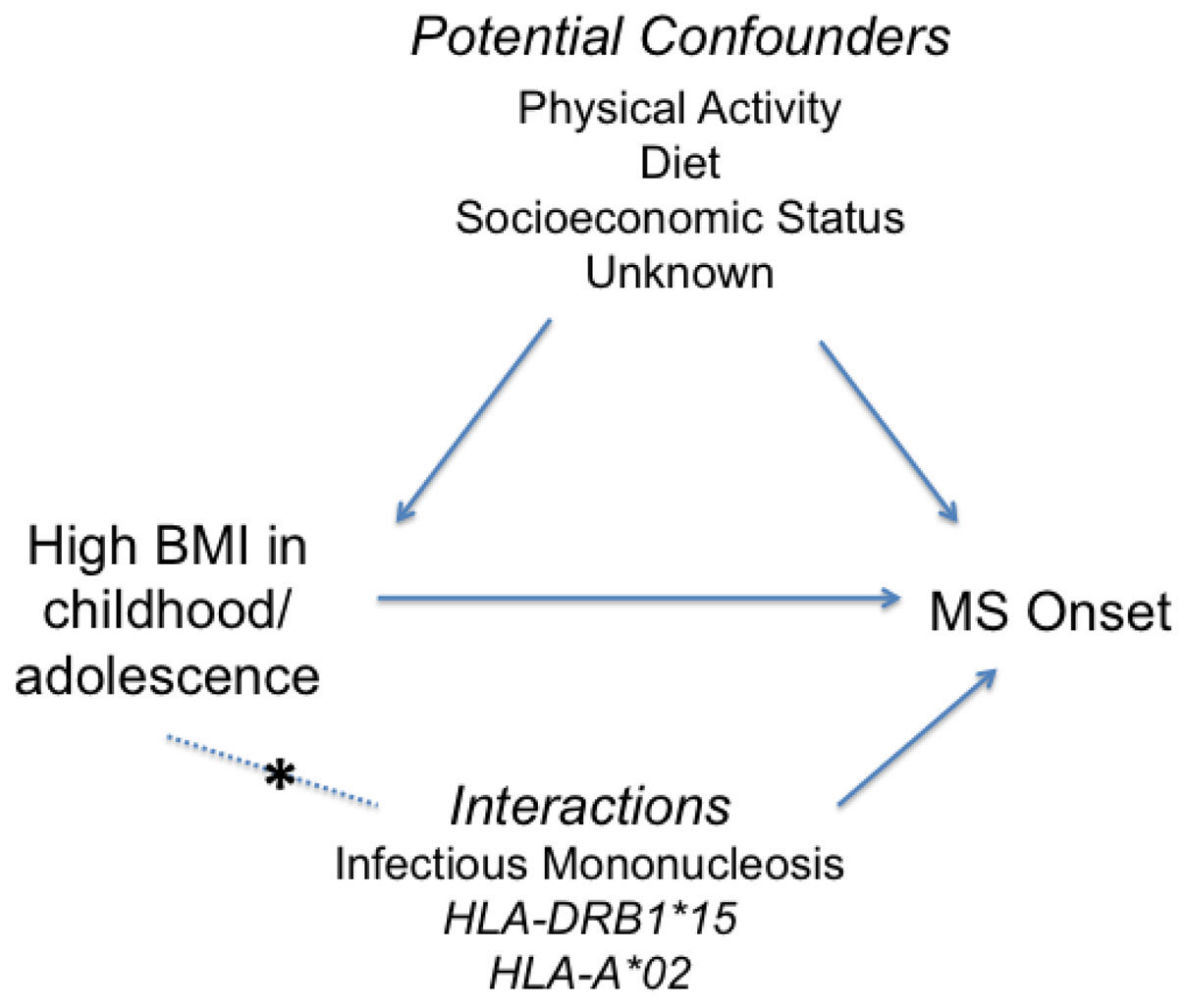
Diagram demonstrating the association between increased body mass index (BMI) and multiple sclerosis (MS) onset, as well as potential confounding factors. Asterisk (*) indicates interaction between BMI and genetic or environmental risk factor.

There is also evidence that obesity interacts with genetic and environmental factors to increase MS susceptibility (Figure 1). In a study that included data from case-control studies in the U.S. and Sweden, having a BMI ≥ 27 kg/m^2^ in young adulthood and carrying 1–2 risk alleles of HLA-DRB1*15 was associated with a seven-fold increased risk of MS compared to non-carriers with a BMI < 27 kg/m^2 23^. A significant interaction was also observed in both populations between having a BMI ≥ 27 kg/m^2^ in young adulthood and carrying 1–2 risk alleles of HLA-A*02 (OR_interaction_=3.4–4.1). This study provides strong evidence that obesity interacts with established MS genetic risk loci, similar to other environmental factors such as smoking^24^ and Epstein-Barr virus exposure^25^ to influence MS susceptibility.

A separate study reported a significant interaction between adolescent BMI and IM, which can be caused by Epstein-Barr virus, associated with MS risk^26^. Individuals who with a BMI > 27 and a history of IM had over a six-fold increased risk of MS compared to individuals with a BMI < 27 without IM. Notably, this was much higher than the risk of MS due to BMI (OR=1.3–1.7) or IM (OR=1.8–2.0) alone.

Future studies examining interactions beyond HLA, such as the >110 non-MHC MS genetic variants^3^ and obesity remain to be investigated, as do interactions between obesity and other established environmental factors, such as smoking and vitamin D level.

## Potential Biological Mechanisms

The biological mechanism underlying the association between obesity and MS is unknown; however, several hypotheses have been proposed. Obesity is characterized by a chronic, low-grade inflammatory response^27^, and integration of metabolic tissue and immune cells contribute to obesity and obesity-related inflammation by sharing a common cellular target^28^. Inflammation in adipose tissue may occur as early as in childhood^29^. Childhood and adolescence obesity is also associated with higher levels of C-reactive protein, interleukin-6, and leptin levels^30–32^, reflecting a proinflammatory state that may influence MS pathogenesis. In fact, serum levels of several adipokines, including leptin, adiponectin and resistin have been found to be associated with autoimmune disease, including MS^33^. Additionally, gut microbiota shape the immune response and may influence inflammation through induction of Th-17 responses^34–36^.

Another hypothesis underscores the role of vitamin D, which has been shown to be a strong risk factor for MS. Adults and children with high body fat mass have lower circulating levels of vitamin D metabolites^37,38^. Lower levels of vitamin D have been associated with increased risk for MS in whites^39^ and more severe disease progression^40^. Thus, overweight and obese individuals may be at particularly high risk for developing MS compared to normal weight individuals. Whether increased obesity leads to a higher risk of MS exclusively through vitamin D deficiency or though some other mechanism is still unknown. Future experimental studies untangling the pathway mediating the relationship between obesity, vitamin D and MS in white and non-white populations are needed.

## Evidence of a Causal Association

Attempts to determine a causal association between obesity and MS susceptibility have recently been conducted^41,42^. Mendelian randomization is a type of instrumental variable (IV) analysis that uses genetic variants strongly associated with an exposure, rather than a direct measure of the exposure, to estimate the effect of the exposure on an outcome. Actual BMI measures are not necessary for the analysis, which is especially useful in the case of a modifiable risk factor such as obesity, which is difficult to measure over a lifetime. Additionally, height and weight can also be biased if self-reported, may vary based on age, and are associated with a wide range of lifestyle and socioeconomic characteristics that may confound a true relationship between obesity and MS. For example, studies have shown that both high and low socioeconomic status (SES) are associated with MS risk^43^, and obesity prevalence also varies based on SES^44^. Since genetic variation is inherited, and therefore present at birth for each individual, ‘genetics’ are not affected by most potential confounding variables or by disease status; the typical confounding present in observational studies is not problematic and reverse causation is unlikely.

One study indicated that obesity is causally associated with MS onset using an IV composed of 97 genetic variants associated with BMI^42^. Authors confirmed the results in two populations, and also found evidence that five BMI-associated genes had a direct effect on MS susceptibility. While females and males demonstrated similar effect sizes, findings were only significant in females, potentially due to a smaller sample size of males. The mechanism of these genetic variants and how they specifically contribute to obesity and disease processes, such as MS, remains to be investigated.

## Conclusion

Strong evidence supports a role for both childhood and adolescence obesity in MS susceptibility, and indicates the relationship may be causal. Results demonstrating a significant causal association offset concerns about potential confounding present in observational studies that may shift estimates away from or towards a null association, such as recall bias with respect to weight, or lack of adjustment for SES in multivariate modeling. While the association has consistently been shown in females, evidence in males is mixed. This may primarily be due to the higher prevalence of MS in females, similar to other autoimmune diseases, and smaller male sample sizes in most studies. Future research should investigate how obesity impacts measures of disease progression in MS over time. Connections between BMI and interferon response^45^, salt intake and other dietary factors^46^, and socioeconomic status^47^ in the context of MS susceptibility have also been studied. Given that obesity is a modifiable risk factor, the importance of its influence on disease onset and progression is critical to reducing the physical, emotional and economic burden of MS in the population.
